# Research on Alloying Elements’ Influence on CuETP-Grade Copper’s Mechanical and Electrical Properties

**DOI:** 10.3390/ma17123020

**Published:** 2024-06-20

**Authors:** Krystian Franczak, Michał Sadzikowski, Paweł Kwaśniewski, Grzegorz Kiesiewicz, Wojciech Ściężor, Szymon Kordaszewski

**Affiliations:** Faculty of Non-Ferrous Metals, AGH University of Krakow, 30-059 Krakow, Poland

**Keywords:** copper, copper alloys, mechanical properties, electrical properties, alloying elements

## Abstract

The continuous industrial development that occurs worldwide generates the need to develop new materials with increasingly higher functional properties. This need also applies to the basic material for electricity purposes, which is copper. In this article, we carry out studies on the influence of various alloying elements such as Mg, In, Si, Nb, Hf, Sb, Ni, Al, Fe, Zr, Cr, Zn, P, Ag, Sc, Pb, Sn, Co, Ti, Mn, Te and Bi on the electrical and mechanical properties of ETP-grade copper. The research involves producing copper alloys using the gravity die casting method with alloy additions of 0.1 wt.%, 0.3 wt.% and 0.5 wt.%. All resulting materials are cold-worked to produce wires, which are subsequently homogenized and annealed. The materials produced in this manner undergo testing to determine their specific electrical conductivity, tensile strength, yield strength, elongation and Vickers hardness (HV10 scale).

## 1. Introduction

**Figure 1:**
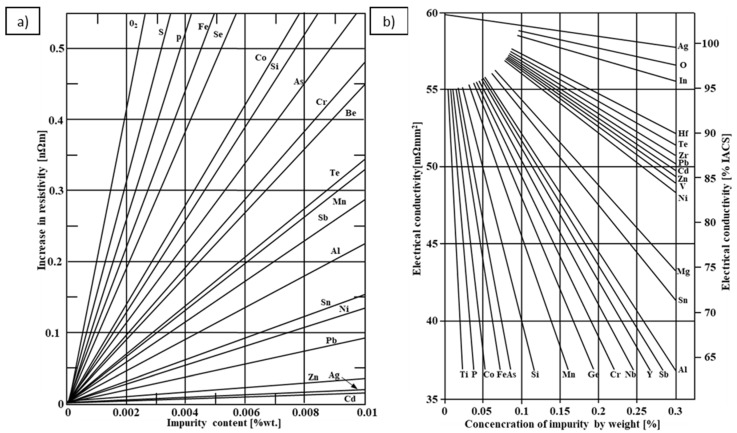
(**a**) Increase in copper electrical resistivity as a function of different impurities’ concentration [13]; (**b**) Influence of various impurities on electrical conductivity (approximated results) [4].

Over the centuries, there has been a continuous development of various methods allowing us to produce materials with increasingly higher functional properties. This also applies to one of the most popular metals, i.e., copper, which is naturally characterized by very high electrical properties and is therefore used mainly for electrical purposes. Copper, apart from silver, has the highest specific electrical conductivity, better than gold and aluminum, but considering the economic aspect (copper is less expensive than silver), the use of copper for electrical purposes is the most justified. However, in its pure form, copper is characterized mainly by a very high plasticity but low mechanical strength, which significantly reduces its application possibilities [1,2,3,4,5]. To increase the functional properties, numerous additions of other elements are used to obtain copper alloys that have a much higher strength compared to pure copper, but also a significantly lower electrical conductivity [6,7]. In 1905, Addikcs published a scientific work in which he presented the influence of individual elements on the electrical conductivity of copper. The cited results showed that the least influence on electrical conductivity of copper (lowest degradation of electrical conductivity) was caused by elements such as Ag, Cd and Zn, while the greatest (highest degradation of electrical conductivity) influence was caused by P, Si and Fe [8]. For many years, detailed research has been carried out on this phenomenon, i.e., the influence of individual elements on the electrical conductivity of copper, and according to the literature, the greatest decrease in conductivity occurs for alloying elements forming solid solutions. A lower decrease is reported for materials subjected to heat treatment through the aging process and for elements present in the copper matrix in the form of a separate phase [9]. In addition, the electrical properties of copper are also influenced by parameters such as the atomic size of foreign elements. Depending on the type of element, the electrical conductivity of copper decreases from the nominal value of 58 MS/m (100% IACS) to a value depending on the type and amount of the alloy addition, as presented in Figure 1 [1,6,10,11,12].

**Figure 2:**
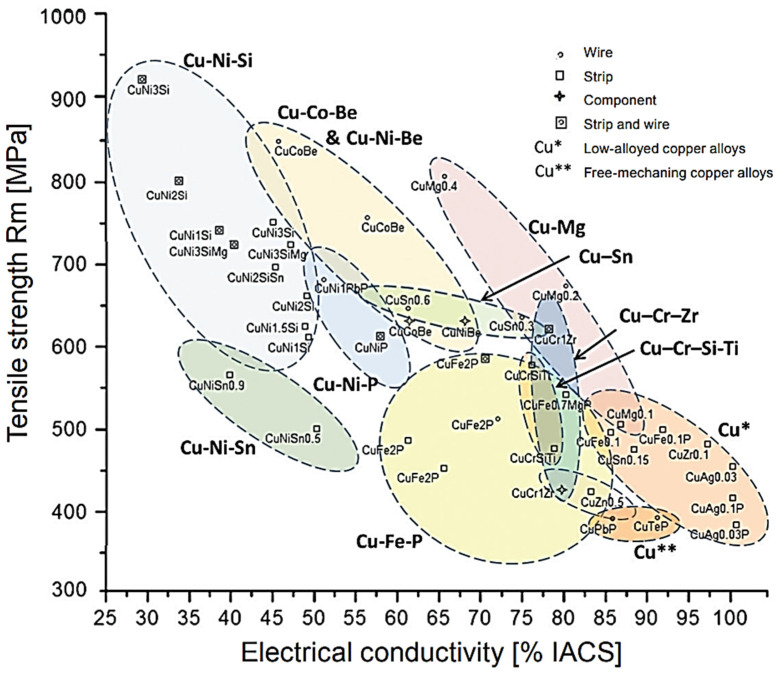
Comparison of mechanical and electrical properties of various copper alloys [1,6,27].

In addition to affecting the electrical properties of copper, alloy additions also strengthen the material. In order to obtain high mechanical properties, several material-strengthening methods can be used including solid solution strengthening, precipitation or dispersion hardening and strain hardening which includes among others grain refinement phenomena [14]. This article uses the precision and distribution hardening method to prepare a super-strong multifunctional coating with mechanical stability. It should also be noted that individual elements will have different effects on the mechanical properties of the obtained copper alloys depending on the strengthening mechanism [15,16,17]. The copper alloys typically used for electrical purposes are alloys containing up to a 1 wt.% addition of other elements, i.e., Cu-Cr, Cu-Zr, Cu-Ag, Cu-Cd, Cu-Mg, Cu-Cr-Zr, Cu-Sn, Cu-Ni-Si, Cu-Ti and Cu-Zn, which are characterized by an electrical conductivity ranging from 58 MS/m down to 5 MS/m and a tensile strength ranging from 180 MPa (for pure copper in a annealed state) to 950 MPa [13,18,19,20,21,22,23,24,25,26,27,28,29,30]. The schematic range of properties of various of the most popular copper alloys is shown in Figure 2.

Copper and its alloys can be produced through the use of various methods; one of the basic ones is the gravity die casting process. This method is used, among other things, to obtain copper in the form of anodes for further processing into cathodes and ready-made elements with simple geometry [31]. This method allows us to obtain casts in a relatively simple manner in contrast with other casting techniques, i.e., centrifugal casting, which makes it possible to obtain higher-quality elements [32]. One of the more advanced methods, allowing us to produce bars, pipes and other semi-finished products, is continuous casting technology. This method can be implemented in either horizontal, vertical upward or downward crystallization direction affecting the final quality and properties of the obtained casts [33]. In addition, a powder metallurgy method is also available; however, it does not involve the melting and crystallization of liquid metal, but only pressing metal from powder of various gradations [34].

It is observed that the current, available literature does not include new research results on the influence of alloying elements on the electrical properties of copper in the range of up to 0.5 wt.%. Therefore, in this article the authors included research on the production of materials and experimental determination of their specific electrical conductivity. Furthermore, an additional aspect of this work are the results of the alloy addition influence on the mechanical properties of copper. The tests included the use of alloying additives such as Mg, In, Si, Nb, Hf, Sb, Ni, Al, Fe, Zr, Cr, Zn, P, Ag, Sc, Pb, Sn, Co, Ti, Mn, Te and Bi in their nominal amount of 0.1 wt.%, 0.3 wt.% and 0.5 wt.% and the reference material in the form of standard CuETP-grade copper.

In the authors’ opinion, all of the research shown has high significance and value, especially in the process of designing new copper-based alloys and new applications for non-ferrous metals field. The obtained test results will enable the selection of appropriate alloying elements to develop new copper alloys with targeted functional properties dedicated for use under high and variable mechanical and electrical loads for the power engineering industry. Specific applications of alloyed copper with improved mechanical and electrical properties include, among others, railway and tramway overhead traction equipment, RSW electrodes for automotive industry, screw connectors and others, where there is a need for both good electrical conductivity and high strength.

## 2. Methods and Materials

**Figure 3:**
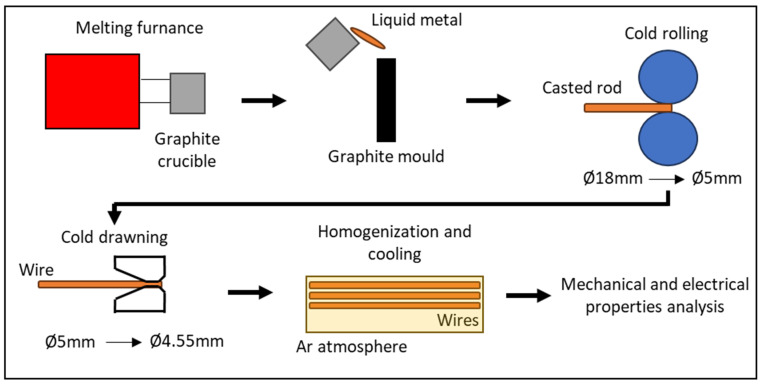
Schematic representation of research program.

The research methodology covers the production of materials through the die casting process, followed by cold rolling and final heat treatment. The ingot casting process was carried out with the use of induction furnace and a commercial graphite crucible. After the heating of copper to 1250–1280 °C (with the use of a graphite surface agent protecting the copper against oxidation), alloy additives were added in their pure metallic form or in the forms of master alloys (see Table 1 and Table 2). Next, after metallurgical synthesis, the liquid metal was poured into a ø20 × 160 mm graphite mold. All the obtained castings were subjected to chemical composition analysis with the use of a reference standard method and spark-optical emission spectrometry (Spectro Spectrotest TX03 (Kleve, Germany)). The obtained castings were next subjected to external-layer machining in order to remove surface imperfections after the crystallization process, and following the cold rolling process, starting from ø18 mm diameter and finishing on ø5 mm. In the last stage of metal forming, in order to improve the surface quality and dimensions, a cold drawing process was used which allowed us to obtain 4.55 mm wires (one-stage cold drawing process). Wires prepared in that manner were subjected to a homogenization process at 800 °C with an inert gas atmosphere (Ar) for 24 h and slowly cooled to ambient temperature at a rate of 0.5 °C/min in the same muffle furnace and also under an Ar gas atmosphere. A schematic diagram showing all of the processes used and described above (allowing us to obtain samples for further research) is presented in Figure 3.

Annealed copper alloy wires were subjected to electrical properties measurements in order to determine the specific electrical conductivity. For this purpose, a four-terminal Thomson bridge method was used (Burster Resistomat 2304 (Gernsbach, Germany)). The resistance (R) measurements were carried out on wires with a length (L) of 1000 mm and a diameter of 4.55 mm (which enabled us to calculate the individual wire’s cross-section (A)), at a temperature of 20 °C. Next, the resistivity (ρ) of the materials was determined based on the following formula:

(1) $$\rho =\frac{R\times A}{L}\;[\Omega m]$$

The obtained research results were recalculated to give the specific electrical conductivity (σ) according to the following equation:

(2) $$\sigma =\frac{1}{\rho }\;[\mathrm{Ms}/m]$$

In the case of materials from which wires could not be made (due to the lack of plasticity), the electrical conductivity was measured using an eddy current instrument (SigmaTest 2.069 (Pittsburgh, PA, USA)).

The mechanical properties of copper alloy wires were analyzed using a Zwick/Roell Z100 (Ulm, Germany) testing machine. The tests were carried out on a 100 mm sample and were conducted at a speed of 50 mm/min with an extensometer. Based on the obtained results, the ultimate tensile strength (UTS)—(3); the yield strength (*YS*)—(4); and the percentage elongation ($E_{A100}$)—(5) were determined according to the following formulas:

(3) $$UTS=\frac{F}{A}\;[\mathrm{MPa}]$$

where

F—maximum applied load in a static tensile test,

A—original specimen cross-sectional area.

(4) $$YS=\frac{F_{0,2}}{A}\;[\mathrm{MPa}]$$

where

$F_{0,2}$—load at which 0.2% plastic deformation occurs during a static tensile test.

(5) $$E_{A100}=\frac{l_{1}-l_{0}}{l_{0}}\times 100\%$$

where

$l_{1}$—increased length of the gauge section after a static tensile test,

$l_{0}$—initial length of the gauge section (100 mm).

Material hardness analyses were carried out using a Tukon2500 hardness tester (Rolling Meadows, IL, USA) based on the Vickers scale. The tests were carried out with the HV10 indenter (loading for 10 s) on a longitudinal section of the tested wires.

All research results presented in the article were based on the statistical average value from individual measurements conducted in accordance with the scheme presented in Table 3.

## 3. Results and Discussion

In the first stage of the research, the chemical composition of the obtained castings was analyzed in terms of individual alloying elements’ content in copper with a nominal content in accordance with Table 4.

Based on chemical composition analysis, it can be stated that the produced copper alloys are characterized by the appropriate contents of alloy additives, which enabled their further experimental analysis to be carried out.

In the next step, tests of the mechanical and electrical properties of all copper alloys were carried out. Figure 4, Figure 5, Figure 6, Figure 7, Figure 8, Figure 9, Figure 10, Figure 11, Figure 12, Figure 13, Figure 14, Figure 15, Figure 16, Figure 17, Figure 18, Figure 19, Figure 20, Figure 21, Figure 22, Figure 23, Figure 24 and Figure 25 present the obtained research results as a function of the main alloying element content.

When analyzing the research presented in the article, first of all, it should be noted that because of the material production process, copper alloys were made in the form of ø 4.55 mm wires with a content of 0.1, 0.3 and 0.5 wt.% with all selected alloying elements except for Te and Bi. These two additives caused cracking of the castings during cold rolling, which indicates limited deformability, which was also documented by the authors of the publications [36,37]. Therefore, these materials were only tested for hardness and electrical conductivity in their annealed cast state.

**Figure 26:**
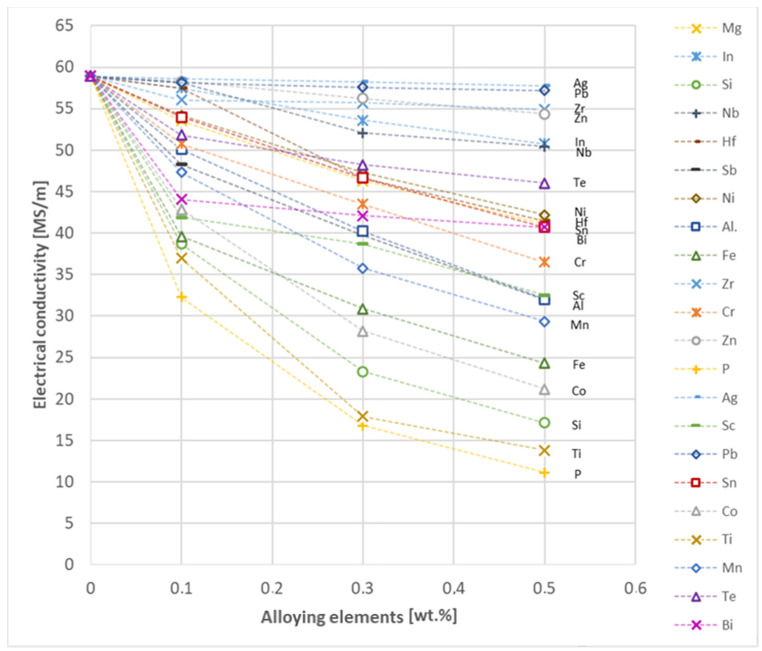
Electrical conductivity as a function of alloying elements’ content in CuETP copper—annealed temper.

An analysis of electrical conductivity shows that the elements having the lowest influence on copper are Ag and Pb at the level above 57.2 MS/m, and Zr and Zn at the level above 54.4 MS/m. The In and Nb additions show electrical conductivity above 50 MS/m. Of all the analyzed elements P, Ti, Si, Co and Fe have the greatest negative impact as an alloying addition to copper—electrical conductivity ranges from 11.2 MS/m up to 24.3 MS/m (see Figure 26). The obtained results in most cases correspond to the results presented in Figure 1b, but due to the different level of alloying element content and the lack of a precisely defined material state in [4,13], the test results cannot be fully compared with existing results.

**Figure 27:**
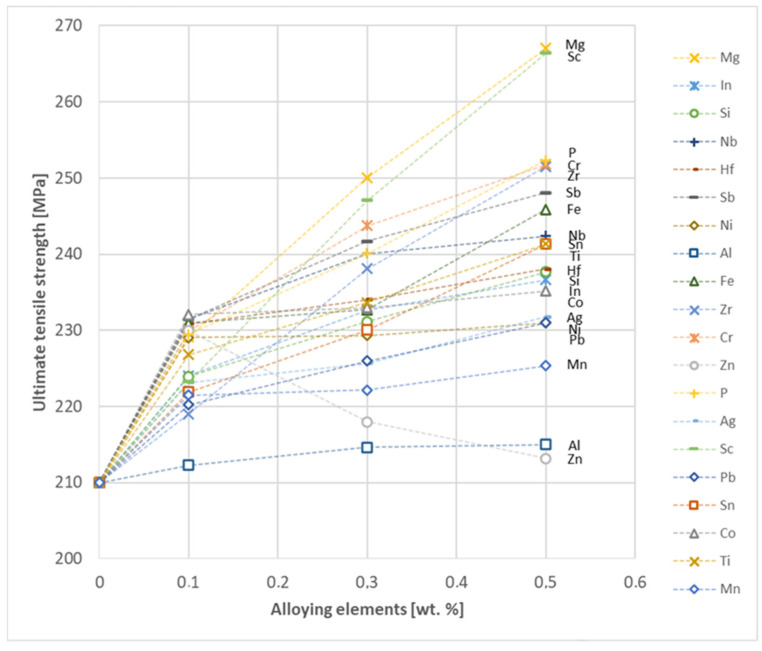
Ultimate tensile strength as a function of alloying elements’ content in CuETP copper—annealed temper.

The tensile strength research results showed that the greatest strengthening of copper comes from the addition of elements such as Mg and Sc, where a content of 0.5 wt.% causes an increase in the UTS of up to 266 MPa. Alloy additives as P, Cr and Zr cause the UTS to increase to the level of 252 MPa. The least strengthening alloy additives (for 0.5 wt.% content) are Zn and Al, which allowed to achieve UTS at the level of 213 MPa and 215 MPa respectively. It is also worth noting that Zn is the only element that reduces the tensile strength of pure copper at their annealed temper (see Figure 27).

**Figure 28:**
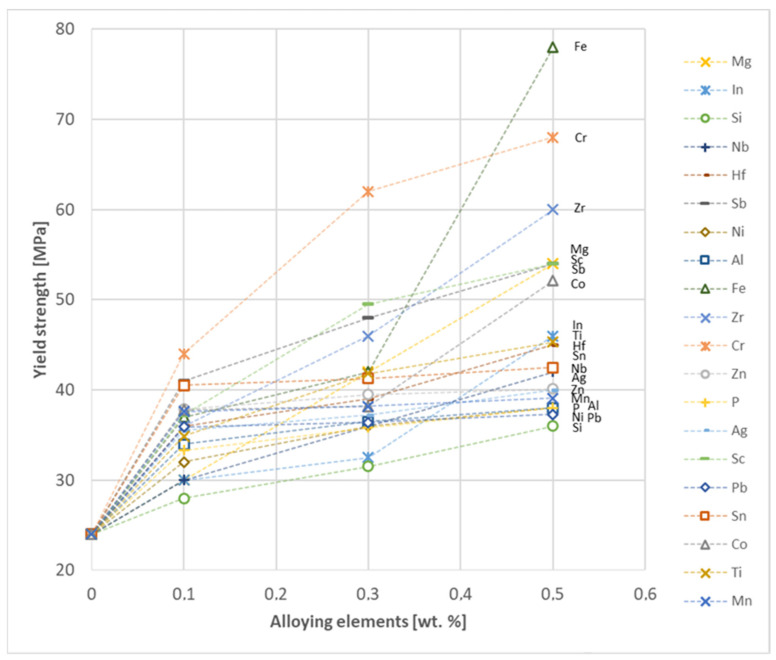
Yield strength as a function of alloying elements’ content in CuETP copper—annealed temper.

The yield strength results showed that the highest increase can be achieved with the use of Fe (78 MPa), Cr (68 MPa) and Zr (60 MPa). The remaining elements, depending on the obtained values, can be divided into two groups: in the range of 52–54 MPa (Mg, Sc, Sb and Co) and 36–46 MPa (other elements), with the lowest influence characterized by Si, Pb and Al addition (Figure 28).

**Figure 29:**
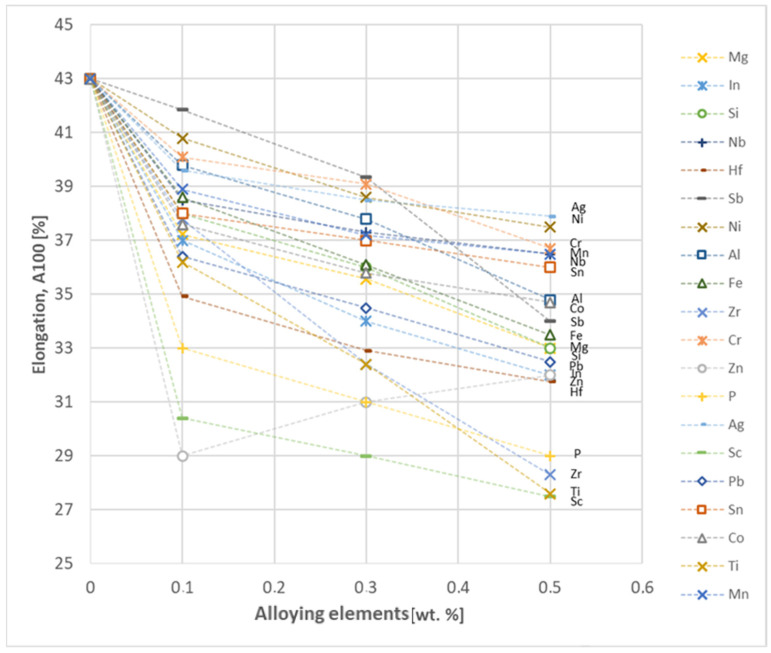
Elongation as a function of alloying elements’ content in CuETP copper—annealed temper.

The obtained elongation results indicate a decrease in the plasticity of copper for all of the selected alloy additives tested in this work. The greatest decrease in elongation from the level of 43% for pure copper was measured for the Sc, Ti, Zr and P elements, oscillating in the range of 27.6–29%. The lowest decrease in the elongation value, for the addition of 0.5 wt.%, was observed for Ag and Ni at the level of 37.5–37.9%. The rest of the alloying additives used during the research were situated within the two above mentioned ranges. Additionally, a reversed tendency in the change in plasticity was observed for Zn—the plasticity of copper increases with the amount of Zn addition from 29% (for 0.1 wt.% of Zn) up to 32% (for 0.5 wt.% of Zn). These results can be correlated with the tensile strength, where this reversed trend was also observed (see Figure 29).

**Figure 30:**
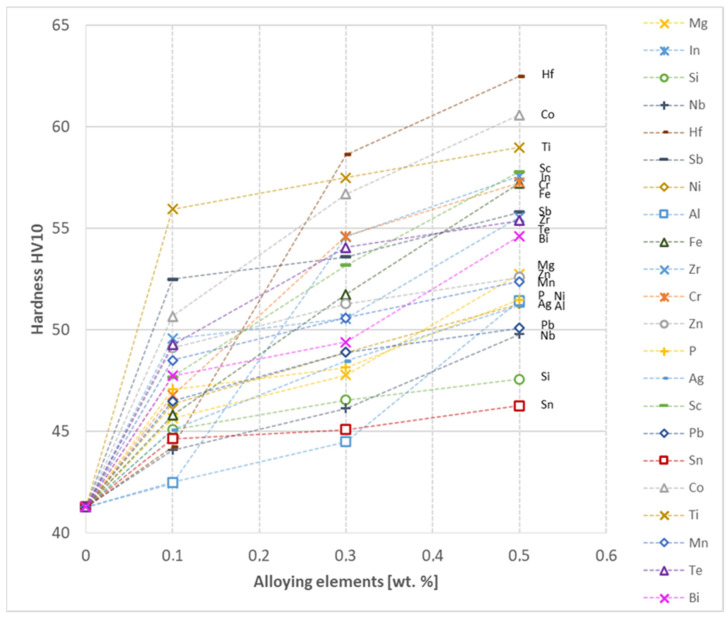
Hardness as a function of alloying elements’ content in CuETP copper—annealed temper.

The hardness analysis presented in the article showed that the greatest increase in the HV10 value can be observed for Hf and Co (62.5 HV10 and 60.6 HV10, respectively) for the 0.5 wt.% element content (the CuETP hardness is at the level of 41.3 HV10). The alloy addition in the form of Ti also significantly increases the hardness of copper, resulting in 59 HV10. The smallest impact on copper hardness was observed for alloy additives such as Sn and Si—the hardness for their highest content (0.5 wt.%) is 46.3 HV10 and 47.6 HV10, respectively. The hardness values of the remaining elements range from 49.8 HV10 up to 57.8 HV10 (see Figure 30).

## 4. Conclusions

Based on the research conducted and presented in the article, the following conclusions were drawn:

1. Copper castings with the addition of Te and Bi, in all their selected contents, cannot be processed with the use of a cold metal forming process, i.e., rolling and drawing, in order to produce wires. This indicates that the cold deformability, due to the addition of Te and Bi, of this alloys is very limited.
2. The electrical conductivity of copper decreases, for the lowest addition of Ag and Pb to the level of 57.2 MS/m and in the case of Zr and Zn to the value of 54.4 MS/m. However, the greatest decrease was observed for P, Ti, Si, Co and Fe addition, where the electrical conductivity ranged from 11.2 MS/m to 24.3 MS/m.
3. The elements that have the greatest impact on the ultimate tensile strength of copper are Mg and Sc—the addition of 0.5 wt.% each increased the CuETP UTS to the level of 266 MPa. However, from all the tested materials, the alloying additions that have the least impact on the tensile strength of copper are Zn and Al, lowering the UTS value to 213 MPa and 215 MPa, respectively.
4. The highest impact on the conventional yield strength of copper was found for Fe, Cr and Zr, which cause the YS to increase to a level slightly above 60 MPa. Si, Pb and Al additions have the lowest impact, with the yield strength being at the level of approximately 36 MPa.
5. The addition of P, Zr, Ti and Sc to CuETP copper causes the plasticity to decrease from the level of 43% (for CuETP) down to 27.6–29%. The smallest impact on the plasticity was observed for Ag and Ni, where A100 elongation was measured at 37.5–37.9%.
6. The highest increase in CuETP hardness was obtained for Hf and Co up to 62.5 HV10 and 60.6 HV10, respectively, both for the 0.5 wt.% content. However, the smallest impact on copper hardness was observed for alloy additions such as Sn and Si, with a resulting hardness, for their highest content, of 46.3 HV10 and 47.6 HV10, respectively.

While this study includes valuable research results of the basic mechanical and electrical properties of copper with various alloying additions, the authors see the potential for future research, especially in the field of work hardening analysis combined with a detailed microstructure analysis of copper-strengthening mechanisms’ aspects.

## Figures and Tables

**Figure 4:**
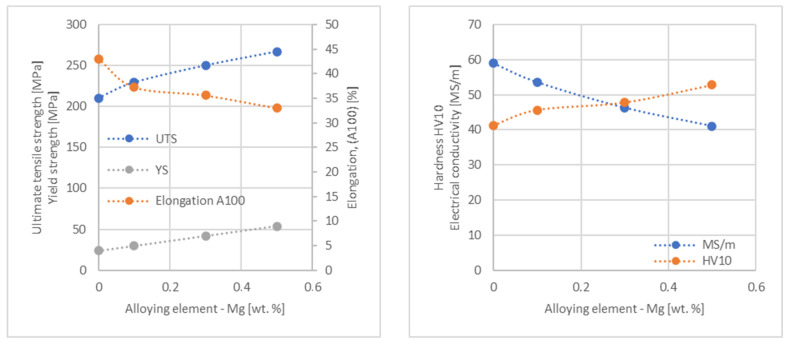
Influence of Mg on copper UTS, YS, elongation (diagram on the **left**), hardness and electrical conductivity (diagram on the **right**).

**Figure 5:**
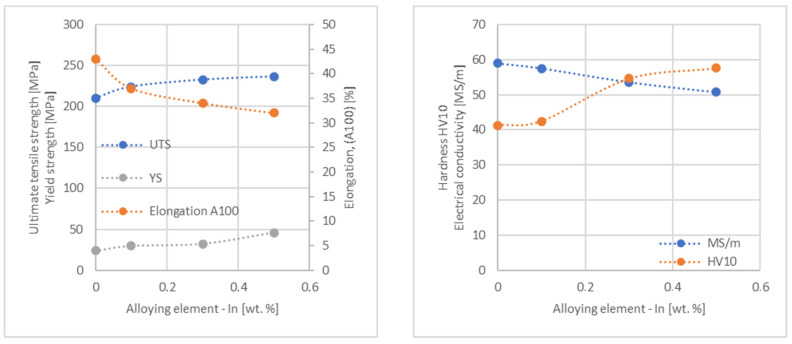
Influence of In on copper UTS, YS, elongation (diagram on the **left**), hardness and electrical conductivity (diagram on the **right**).

**Figure 6:**
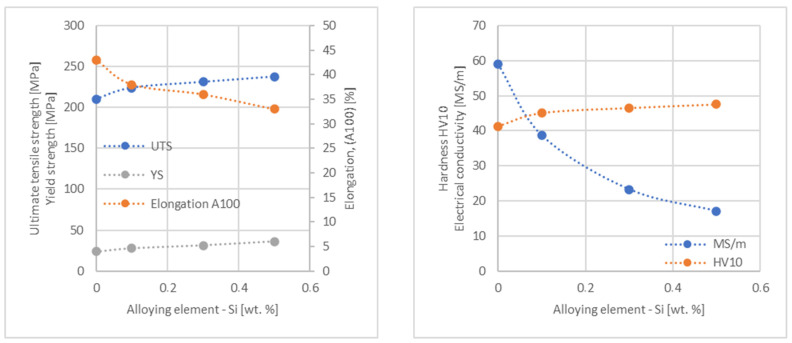
Influence of Si on copper UTS, YS, elongation (diagram on the **left**), hardness and electrical conductivity (diagram on the **right**).

**Figure 7:**
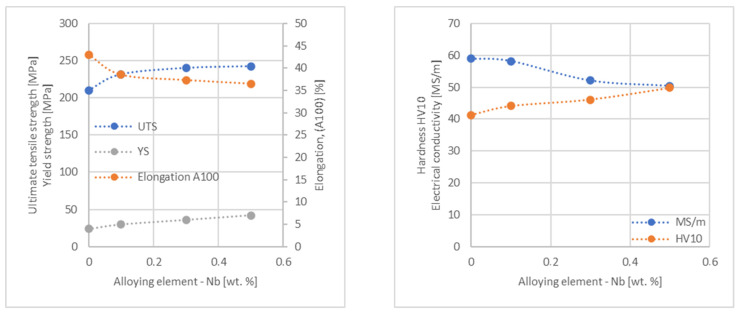
Influence of Nb on copper UTS, YS, elongation (diagram on the **left**), hardness and electrical conductivity (diagram on the **right**).

**Figure 8:**
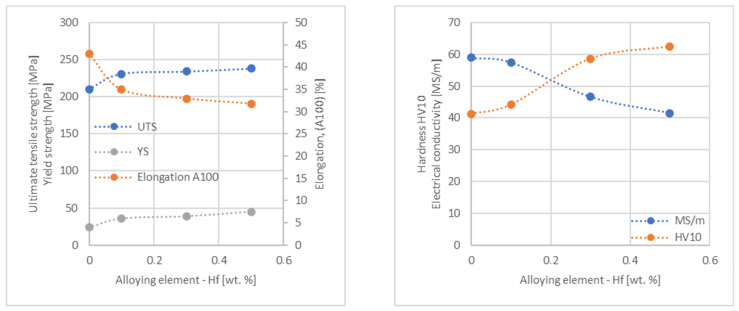
Influence of Hf on CuETP copper UTS, YS, elongation (diagram on the **left**), hardness and electrical conductivity (diagram on the **right**).

**Figure 9:**
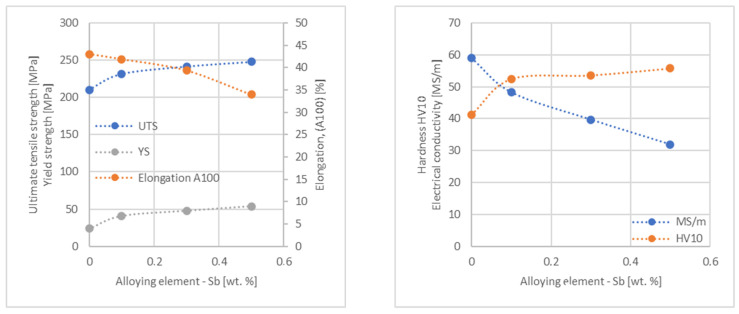
Influence of Sb on CuETP copper UTS, YS, elongation (diagram on the **left**), hardness and electrical conductivity (diagram on the **right**).

**Figure 10:**
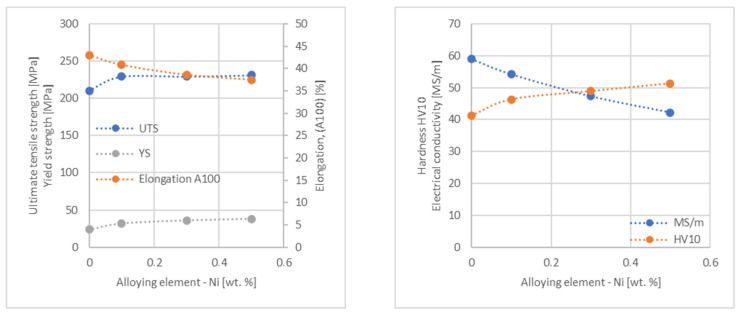
Influence of Ni on CuETP copper UTS, YS, elongation (diagram on the **left**), hardness and electrical conductivity (diagram on the **right**).

**Figure 11:**
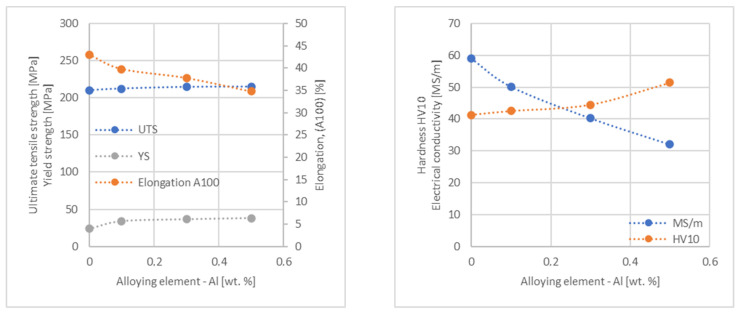
Influence of Al on CuETP copper UTS, YS, elongation (diagram on the **left**), hardness and electrical conductivity (diagram on the **right**).

**Figure 12:**
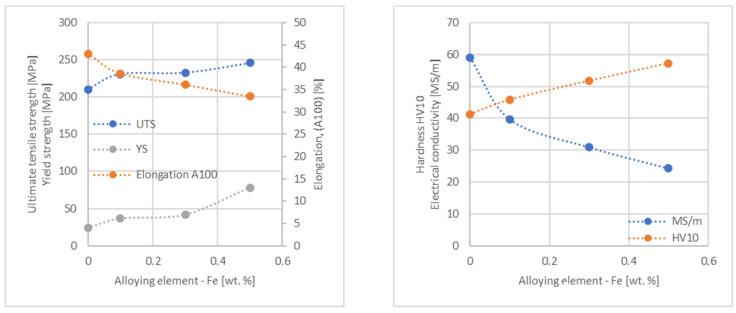
Influence of Fe on CuETP copper UTS, YS, elongation (diagram on the **left**), hardness and electrical conductivity (diagram on the **right**).

**Figure 13:**
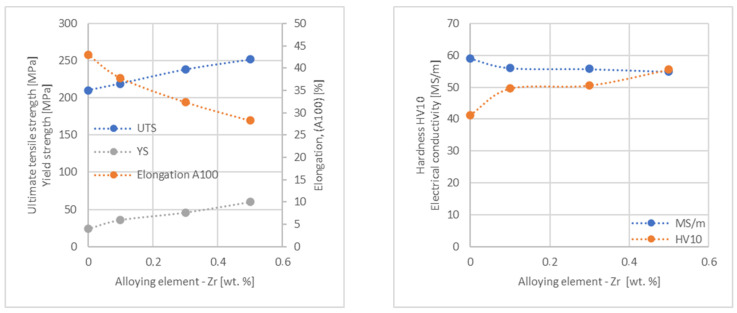
Influence of Zr on CuETP copper UTS, YS, elongation (diagram on the **left**), hardness and electrical conductivity (diagram on the **right**).

**Figure 14:**
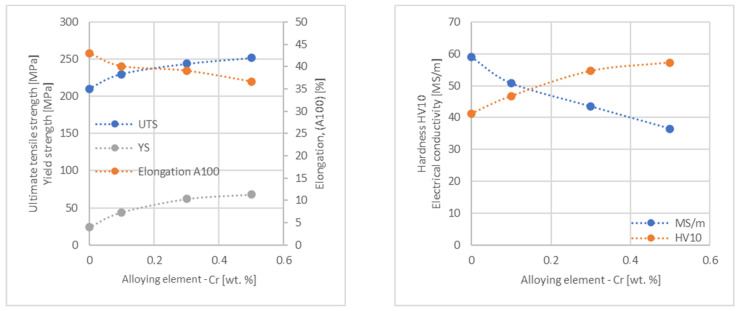
Influence of Cr on CuETP copper UTS, YS, elongation (diagram on the **left**), hardness and electrical conductivity (diagram on the **right**).

**Figure 15:**
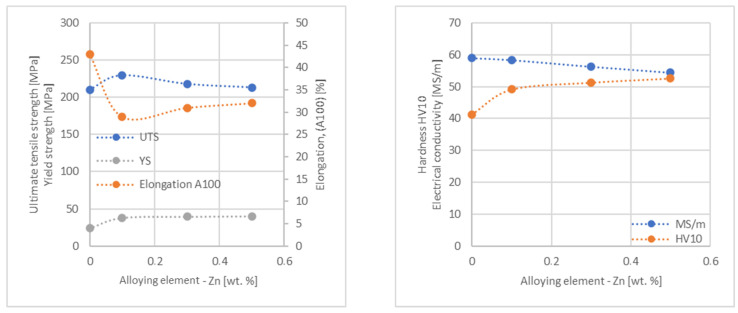
Influence of Zn on CuETP copper UTS, YS, elongation (diagram on the **left**), hardness and electrical conductivity (diagram on the **right**).

**Figure 16:**
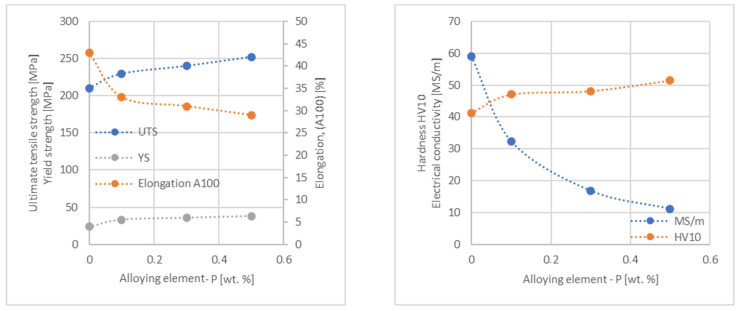
Influence of P on CuETP copper UTS, YS, elongation (diagram on the **left**), hardness and electrical conductivity (diagram on the **right**).

**Figure 17:**
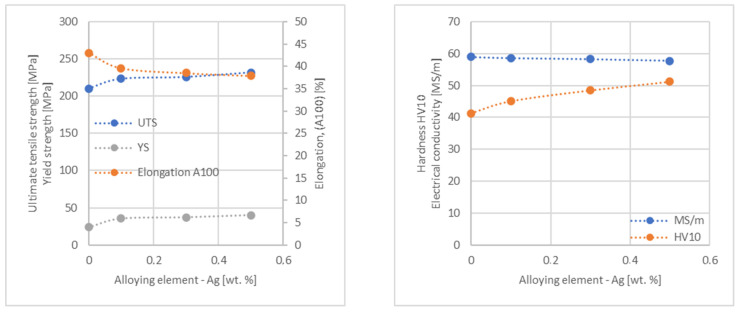
Influence of Ag on CuETP copper UTS, YS, elongation (diagram on the **left**), hardness and electrical conductivity (diagram on the **right**).

**Figure 18:**
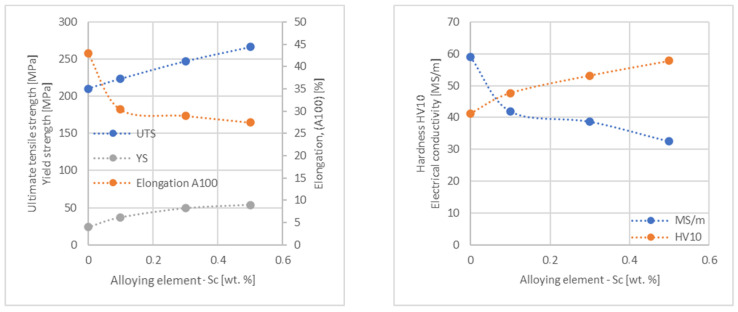
Influence of Sc on CuETP copper UTS, YS, elongation (diagram on the **left**), hardness and electrical conductivity (diagram on the **right**).

**Figure 19:**
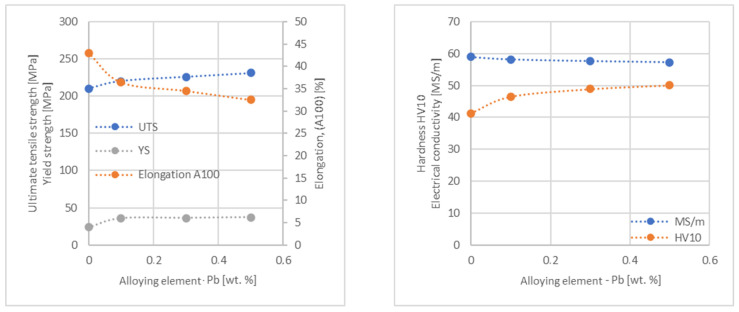
Influence of Pb on CuETP copper UTS, YS, elongation (diagram on the **left**), hardness and electrical conductivity (diagram on the **right**).

**Figure 20:**
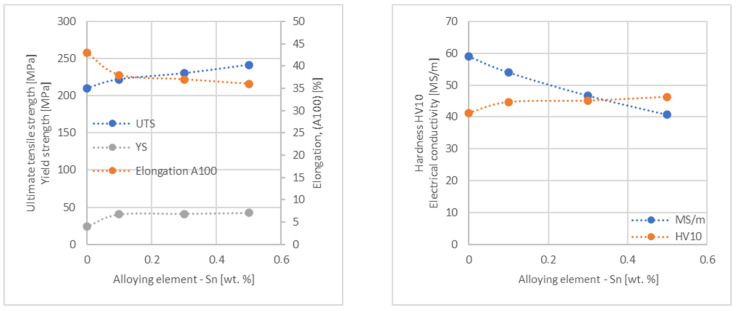
Influence of Sn on CuETP copper UTS, YS, elongation (diagram on the **left**), hardness and electrical conductivity (diagram on the **right**).

**Figure 21:**
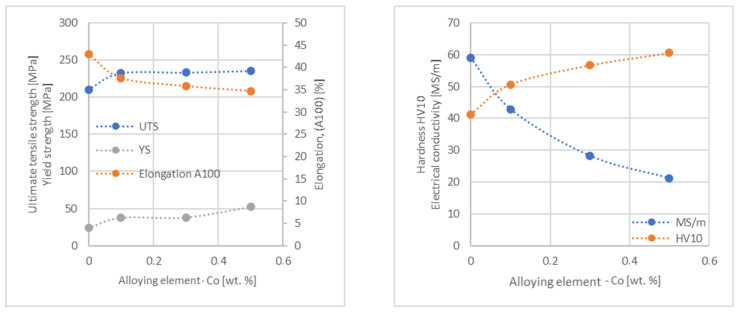
Influence of Co on CuETP copper UTS, YS, elongation (diagram on the **left**), hardness and electrical conductivity (diagram on the **right**).

**Figure 22:**
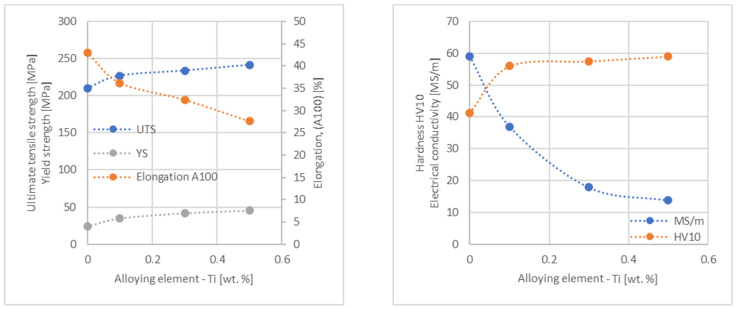
Influence of Ti on CuETP copper UTS, YS, elongation (diagram on the **left**), hardness and electrical conductivity (diagram on the **right**).

**Figure 23:**
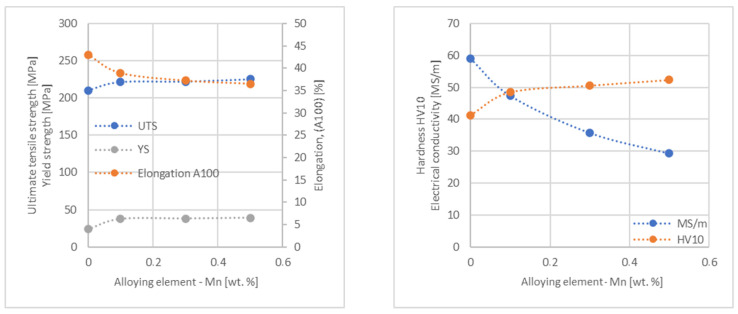
Influence of Mn on CuETP copper UTS, YS, elongation (diagram on the **left**), hardness and electrical conductivity (diagram on the **right**).

**Figure 24:**
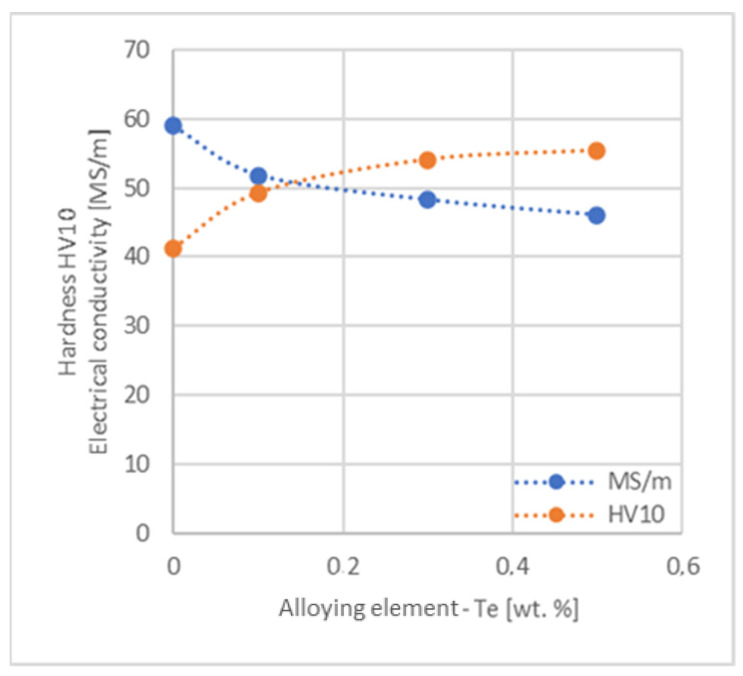
Influence of Te on CuETP copper hardness and electrical conductivity.

**Figure 25:**
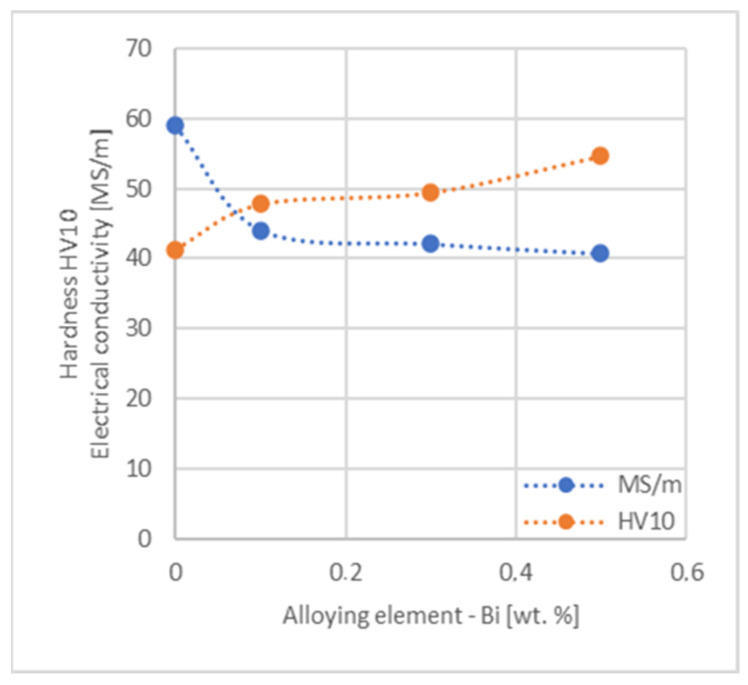
Influence of Bi on CuETP copper hardness and electrical conductivity.

**Table 1:** Chemical composition of CuETP—EN 13601:2021 [35].

Material		Element [ppm]																						Not Included
Alloy	Symbol	Range	Cu *	Ag	As	Bi	Cd	Co	Cr	Fe	Mn	Ni	P	Pb	S	Sb	Se	Si	Sn	Te	Zn	O	Sum	
Cu-ETP1	CW003A	min.	-	-	-	-	-	-	-	-	-	-	-	-	-	-	-	-	-	-	-	-	-	O
		max.	-	25	5 ^(1)^	2 ^(2)^	- ^(2)^	- ^(3)^	- ^(1)^	10 ^(3)^	- ^(1)^	- ^(3)^	- ^(1)^	5	15 ^(4)^	4 ^(1)^	2 ^(2)^	- ^(3)^	- ^(3)^	2 ^(2)^	- ^(3)^	400 ^(6)^	65	

^(1)^ As + Cd + Cr + Mn + P + Sb = max. 0.0015%. ^(2)^ Bi + Se + Te = max. 0.0003%, where Se + Te = max. 0.00030%. ^(3)^ Co + Fe + Ni + Si + Sn + Zn = max. 0.0020%. ^(4)^ Sulphur content should be determined on the casted sample. ^(6)^ Oxygen content should be controlled by the manufacturer to meet hydrogen embrittlement requirements. * remainder.

**Table 2:** List of alloying elements used for metallurgical synthesis.

Pure Metallic Form *	Master Alloy
Mg, In, Si, Nb, Hf, Sb, Ni, Al, Fe, Zn, Ag, Sc, Pb, Sn, Co, Ti, Te, Bi	Cr–CuCr7
	Zr–CuZr10
	Mn–CuMn50
	P–CuP8

* min. 99.9%.

**Table 3:** Research statistics admitted for different properties’ analysis.

Research Type	Test Stand	Number of Measurements/Samples	Final Result
Electrical conductivity	Burster Resistomat 2304 (Gernsbach, Germany)	5	Arithmetic average
	Foerster SigmaTest 2.069 (Pittsburgh, PA, USA)	10	
UTS, YS, elongation	Zwick/Roell Z100 (Ulm, Germany)	5	
Hardness	Wilson Tukon 2500 (Rolling Meadows, IL, USA)	10	
Chemical composition analysis	Spectro Spectrotest TX03 (Kleve, Germany)	5	

**Table 4:** Cumulative summary of all tested samples and main alloying element content in all obtained copper alloys.

Material and Designated Chemical Composition		Tested Main Alloying Element Content	Sample Type	Material and Designated Chemical Composition		Tested Main Alloying Element Content	Sample Type
Cu-Mg	CuMg0.1	0.11	Wire ø4.55 mm	Cu-Cr	CuCr0.1	0.12	Wire ø4.55 mm
	CuMg0.3	0.30			CuCr0.3	0.33	
	CuMg0.5	0.53			CuCr0.5	0.56	
Cu-In	CuIn0.1	0.10		Cu-Zn	CuZn0.1	0.12	
	CuIn0.3	0.29			CuZn0.3	0.31	
	CuIn0.5	0.51			CuZn0.5	0.51	
Cu-Si	CuSi0.1	0.12		Cu-P	CuP0.1	0.14	
	CuSi0.3	0.31			CuP0.3	0.46	
	CuSi0.5	0.49			CuP0.5	0.54	
Cu-Nb	CuNb0.1	0.11		Cu-Ag	CuAg0.1	0.11	
	CuNb0.3	0.33			CuAg0.3	0.32	
	CuNb0.5	0.48			CuAg0.5	0.53	
Cu-Hf	CuHf0.1	0.09		Cu-Sc	CuSc0.1	0.12	
	CuHf0.3	0.27			CuSc0.3	0.31	
	CuHf0.5	0.49			CuSc0.5	0.55	
Cu-Sb	CuSb0.1	0.10		Cu-Pb	CuPb0.1	0.12	
	CuSb0.3	0.26			CuPb0.3	0.33	
	CuSb0.5	0.46			CuPb0.5	0.51	
Cu-Ni	CuNi0.1	0.09		Cu-Sn	CuSn0.1	0.11	
	CuNi0.3	0.30			CuSn0.3	0.31	
	CuNi0.5	0.50			CuSn0.5	0.54	
Cu-Al	CuAl0.1	0.08		Cu-Co	CuCo0.1	0.08	
	CuAl0.3	0.27			CuCo0.3	0.27	
	CuAl0.5	0.46			CuCo0.5	0.46	
Cu-Fe	CuFe0.1	0.07		Cu-Ti	CuTi0.1	0.09	
	CuFe0.3	0.28			CuTi0.3	0.28	
	CuFe0.5	0.45			CuTI0.5	0.47	
Cu-Zr	CuZr0.1	0.08		Cu-Mn	CuMn0.1	0.11	
	CuZr0.3	0.26			CuMn0.3	0.31	
	CuZr0.5	0.48			CuMn0.5	0.49	
Cu-Te	CuTe0.1	0.11	Cast ø18 mm	Cu-Bi	CuBi0.1	0.13	Cast ø18 mm
	CuTe0.3	0.30			CuBi0.3	0.33	
	CuTe0.5	0.51			CuBi0.5	0.51	

## Data Availability

The original contributions presented in the study are included in the article, further inquiries can be directed to the corresponding author.
